# Crystal structure of *catena*-poly[[copper(II)-μ_2_-salicylato-[diaqua­copper(II)]-μ_2_-salicylato] dihydrate]

**DOI:** 10.1107/S2056989017000883

**Published:** 2017-01-20

**Authors:** Jitschaq A. van der Horn, Martin Lutz

**Affiliations:** a Bijvoet Center for Biomolecular Research, Crystal and Structural Chemistry, Faculty of Science, Utrecht University, Padualaan 8, 3584 CH Utrecht, The Netherlands

**Keywords:** crystal structure, copper, salicylate, hydrogen bonding

## Abstract

The title compound, {[Cu_2_(C_7_H_4_O_3_)_2_(H_2_O)_2_]·2H_2_O}_*n*_, contains two copper(II) cations in special positions (one on a twofold rotation axis and one on an inversion centre) and the the salicylate ligand in its dianionic form. By four- and six-coordinate metal coordination, chains are formed parallel to [001], which are extended by O—H⋯O hydrogen bonding into sheets extending parallel to (100). These sheets are weakly connected by O—H⋯O hydrogen bonding *via* the non-coordinating lattice water mol­ecules into a three-dimensional network.

## Abstract 2

Two independent copper(II) cations with coordination numbers of 4 and 6 are bridged by dianionic salicylate anions into chains extending parallel [001]. O—H⋯O hydrogen-bonding inter­actions involving both the coordinating and the lattice water mol­ecules result in the formation of a three-dimensional network.

## Chemical context   

Salicylic acid (2-hy­droxy­benzoic acid, H_2_Sal) has two acidic hydrogen atoms and the corresponding p*K*_a_ values are 2.853 (9) and 12.897 (7) (Farajtabar & Gharib, 2010 ▸; García *et al.*, 1982 ▸). Titration studies with Cu^2+^ indeed indicate the formation of complexes with the monoanionic ligand HSal^−^ as well as with the dianionic ligand Sal^2−^ (Dahlund & Olin, 1988 ▸; Furia & Porto, 2002 ▸). From the literature, crystal structures of copper salicylate are only known with the monoanionic HSal^−^ ligand. They occur as a tetra­hydrate (Hanic & Michalov, 1960 ▸; Rissanen *et al.*, 1987 ▸) and as a dihydrate (Jagner *et al.*, 1976 ▸), the latter being described as an order–disorder structure. In an attempt to crystallize copper(II) salicylate we obtained a mixture of crystals (see *Synthesis and Crystallization*), among which was the title compound (I) with composition [Cu_2_(C_7_H_4_O_3_)_2_(H_2_O)_2_]·2H_2_O that involves the dianionic ligand Sal^2−^.
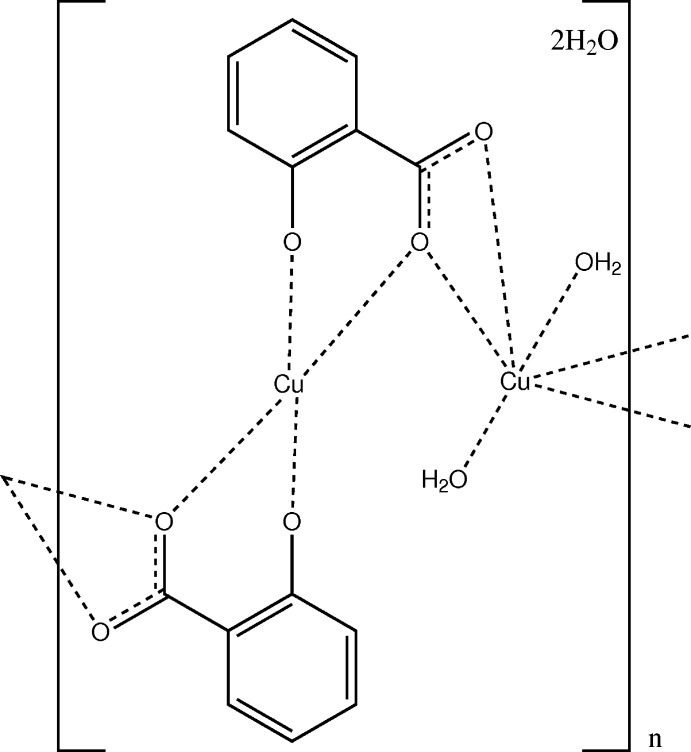


## Structural commentary   

**Figure 1:**
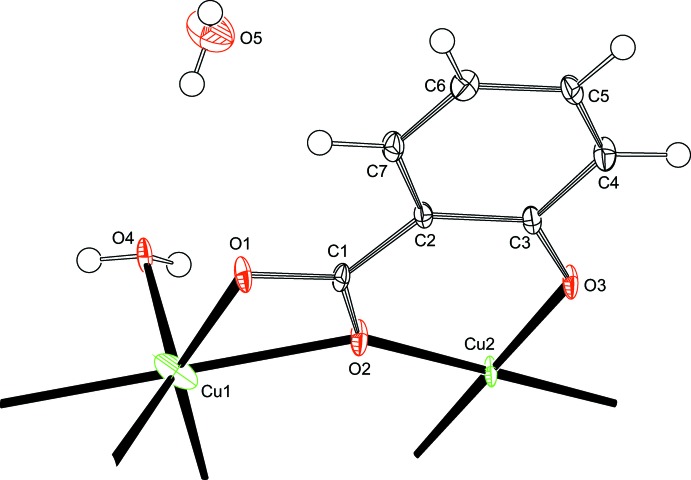
The asymmetric unit in the crystal structure of (I) in a view along [010]. Displacement ellipsoids are drawn at the 50% probability level. H atoms are drawn as spheres with an arbitrary radius. Additional bonds to O atoms outside the asymmetric unit indicate the completed coordination environments of the two copper(II) cations.

**Figure 2:**
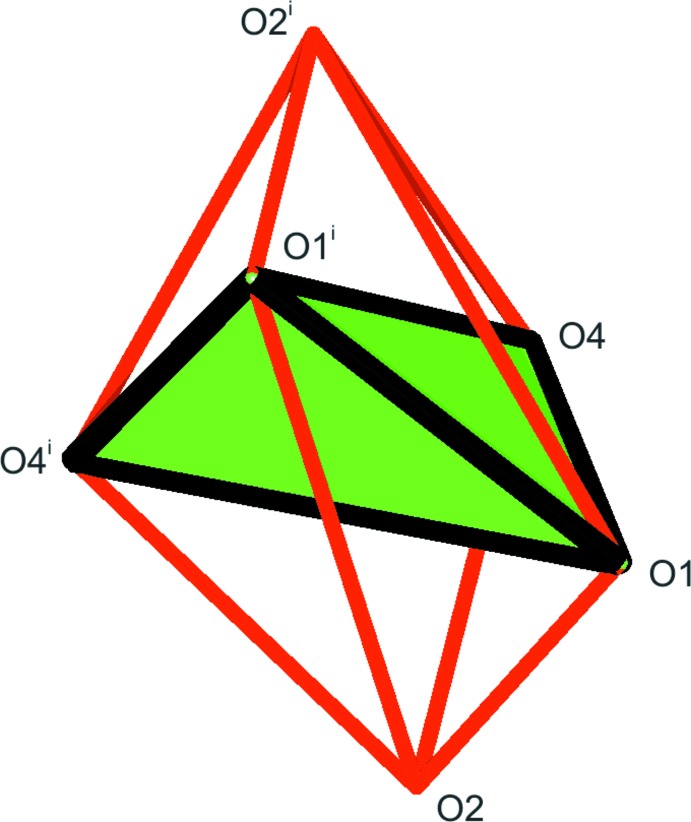
The environment of the Cu1 atom with *C*_2_ symmetry. Because the Cu1—O2 distance is rather long, the metal atom can either be described as four-coordinated (green) or as six-coordinated (red). In both cases, the coordination geometry is severely distorted. [Symmetry code: (i) 1 − *x*, *y*, 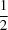 − *z*.]

The asymmetric unit of (I) is shown in Fig. 1 ▸. The two copper(II) cations are located on special positions with twofold rotation symmetry (Cu1, Wyckoff position *c*) and inversion symmetry (Cu2, Wyckoff position *b*). Cu2 is four-coordinated in a square-planar configuration with donor atoms O2 of the carboxyl­ate and O3 of the deprotonated hy­droxy group. The two pairs of Cu—O distances are 1.905 (2) Å and are the shortest in the present structure (Table 1 ▸). As a consequence of the inversion symmetry, the fourfold coordination environment is exactly planar with an angle sum of 360.0 (2)°. Cu1 has an environment of six oxygen atoms (Fig. 2 ▸). The Cu1—O1 distance to a carboxyl­ate oxygen atom, and the Cu1—O4 distance to the coordinating water mol­ecule are in the expected range. The Cu1—O2 distance of 2.332 (2) Å is rather long, which indicates only a weak inter­action. The twofold rotation axis bis­ects the O1—Cu1—O1^i^ and the O4—Cu1—O4^i^ angles [symmetry code: (i) 1 − *x*, *y*, 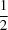 − *z*]. This allows the five atoms Cu1, O1, O4, O1^i^ and O4^i^ to deviate significantly from planarity. The sum of the *cis* angles is 382.8 (3)° and the dihedral angle between the O1—Cu1—O1^i^/O4—Cu1—O4^i^ planes is 49.86 (14)°. If one decides to consider Cu1 as four-coordinated, the coordination environment is consequently best described as halfway between square-planar and tetra­hedral with approximate *D*_2*d*_ symmetry [*τ*_4_ parameter = 0.52; *θ*_6_ = 94.60 (11)°; Yang *et al.*, 2007 ▸]. The O2—Cu1—O2^i^ angle is nearly perpendicular to the twofold axis and thus at 176.45 (12)° nearly linear. A description as a six-coordinated metal cation can nevertheless only be called very distorted due to the non-planarity of the equatorial atoms.

The Cu1—O2 bond fails the Hirshfeld rigid-bond test (Hirshfeld, 1976 ▸) with Δ m.s.d.a. of 0.0200 (13) Å^2^ as calculated with the *PLATON* software (Spek, 2009 ▸). A similar effect has been observed in bidentate Zn—O(carboxyl­ate) complexes and was attributed to the strain in the four-membered chelate ring (Lutz & Spek, 2009 ▸). In the present case, it can also be ascribed to the weakness of the inter­action, which allows a rather uncorrelated movement of Cu1 and O2. In fact, O2 is bridging between Cu1 and Cu2 and the O2—Cu2 bond is much stronger than O2—Cu1. Δ m.s.d.a. for O2—Cu2 is only 0.0007 (13) Å^2^ and inconspicuous.

The salicylate dianion is located on a general position. It is essentially planar with a maximum deviation of 0.054 (3) Å from the least-squares plane. This small deviation involves the carboxyl­ate group with torsion angles of −3.4 (5) ° for C7—C2—C1—O1 and −2.5 (6) ° for C3—C2—C1—O2. The C—OH bond length of 1.359 (2) Å in Cu(HSal)_2_·4H_2_O (Rissanen *et al.*, 1987 ▸) is shortened to 1.322 (4) Å after deprotonation in the present compound [Cu_2_(C_7_H_4_O_3_)_2_(H_2_O)_2_]·2H_2_O (Table 1 ▸). One of the water mol­ecules (O4) coordinates to the Cu1 copper(II) ion, while the other water mol­ecule (O5) is present as non-coordinating lattice water.

## Supra­molecular features   

**Figure 3:**
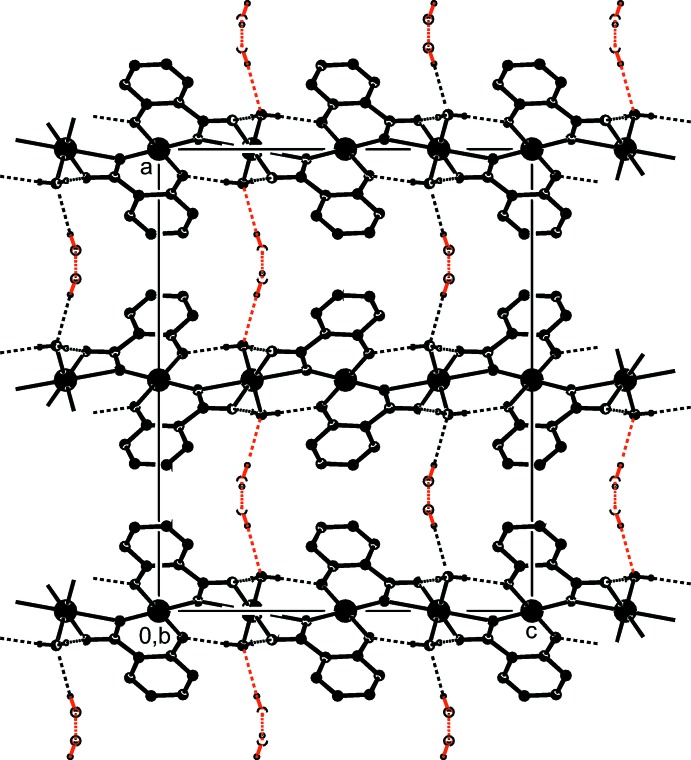
The packing of (I) in the crystal, in a view along [010], showing the hydrogen-bonded layers (black) parallel to (100). The layers are linked *via* non-coordinating hydrate water mol­ecules (red) into a three-dimensional network. C—H hydrogen atoms have been omitted for clarity.

Compound (I) forms coordination chains extending parallel to [001] with a Cu⋯Cu distance of 4.0478 (4) Å. The coordinating water mol­ecule O4 acts as a donor of hydrogen bonds with the carboxyl­ate oxygen O1 and the deprotonated hy­droxy oxygen O3 as acceptors (Table 2 ▸). This extends the one-dimensional coordination polymer into a two-dimensional hydrogen-bonded network parallel to (100) (Fig. 3 ▸). Between the hydrogen-bonded layers there are solvent-accessible channels along [010] at the positions *x* = 0.25 and *z* = 0.25, which is the inter­section of two glide planes. By symmetry, there are four channels per unit cell with a volume of 59 Å^3^ each, as calculated with the *PLATON* software (Spek, 2009 ▸). Each channel is occupied by two non-coordinating water mol­ecules O5 per unit cell. The O5 water mol­ecules are linked to each other by cooperative hydrogen bonding, forming chains along [010]. A second hydrogen bond for O5 involves the coordinating water mol­ecule O4. The lattice water mol­ecules thus connect the described (100) layers into a three-dimensional hydrogen-bonded network. It should be noted that the hydrogen bonds O5⋯O4 are rather long (Table 2 ▸) and therefore the link between the layers appears to be weak.

**Figure 4:**
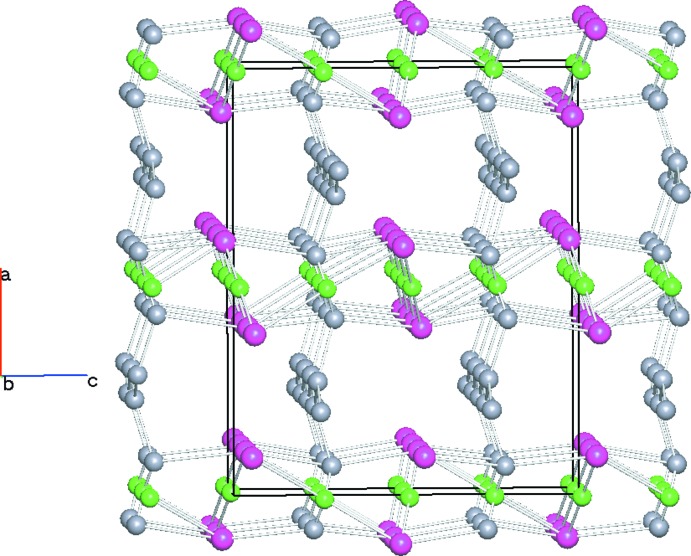
Simplified net as prepared with the *TOPOS* software (Blatov *et al.*, 2014 ▸). Copper cations are shown in green. Nodes derived from the salicylate dianion are displayed in purple and have a coordination number of four. Nodes derived from the water mol­ecules are drawn in grey and have coordination numbers of three and four for the lattice and coordinating water mol­ecules, respectively.

In a more systematic approach the packing can be subjected to a topological analysis using *TOPOS* (Blatov *et al.*, 2014 ▸). In this process, mol­ecular entities are abstracted as nodes. Cu1 is a node with a coordination number of 4 (linked to two salicylate ligands and two water mol­ecules). Cu2 has a coordination number of 2 (two salicylate ions). The salicylate ion has four neighbours (two copper ions and two hydrogen bonds). Water mol­ecule O4 is connected to four nodes (one copper ion and three hydrogen bonds). The lattice water O5 has a coordination number of 3 (three hydrogen bonds). A plot of the simplified structure is given in Fig. 4 ▸.

## Synthesis and crystallization   

0.55 g (4 mmol) salicylic acid were suspended in 8 ml water. With a concentrated NaOH solution the pH value was adjusted to approximately 5. A solution of 0.5 g (2 mmol) copper(II) sulfate penta­hydrate in 10 ml water was added. Crystals appeared after a few days of standing. From the unit-cell determinations it became clear that the mixture of crystals contained at least three species: colourless salicylic acid, green Cu(HSal)_2_·2H_2_O, and brown crystals of (I). The crystals of (I) are thin plates with <100> being the small dimension. A possible explanation for the form is a two-dimensional hydrogen-bonded network in the structure which extends parallel to (100), as discussed above.

## Refinement   

Crystal data, data collection and structure refinement details are summarized in Table 3 ▸.

The diffraction data appeared to contain reflections of a small second crystal fragment related by a *ca* 2° rotation about *hkl* = (4
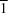
3) with respect to the main fragment. Two orientation matrices were used for the integration with the *Eval15* software (Schreurs *et al.*, 2010 ▸). A large isotropic mosaicity of 1.4° was assumed for the prediction of the reflection profiles. Only the non-overlapping reflections were used for structure solution and refinement.

All hydrogen atoms were located in difference Fourier maps. C—H hydrogen atoms were refined with a riding model. O—H hydrogen atoms were kept fixed at their located positions.

## Supplementary Material

Crystal structure: contains datablock(s) I, global. DOI: 10.1107/S2056989017000883/wm5360sup1.cif

Structure factors: contains datablock(s) I. DOI: 10.1107/S2056989017000883/wm5360Isup2.hkl

CCDC reference: 1528159

Additional supporting information:  crystallographic information; 3D view; checkCIF report

## Figures and Tables

**Table 1:** Selected geometric parameters (Å, °)

Cu1—O1	1.984 (2)	Cu2—O2	1.905 (2)
Cu1—O4	2.001 (3)	O1—C1	1.292 (4)
Cu1—O2	2.332 (2)	O2—C1	1.274 (4)
Cu2—O3	1.905 (2)	O3—C3	1.322 (4)
			
O1—Cu1—O1^i^	96.07 (14)	O3—Cu2—O2	92.62 (10)
O1—Cu1—O4	94.60 (11)	C1—O2—Cu2	129.6 (2)
O4^i^—Cu1—O4	97.54 (15)	C1—O2—Cu1	84.86 (19)
O2—Cu1—O2^i^	176.45 (12)	Cu2—O2—Cu1	145.44 (13)
			
O1—C1—C2—C7	−3.4 (5)	O2—C1—C2—C3	−2.5 (6)

Symmetry code: (i) 
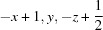
.

**Table 2:** Hydrogen-bond geometry (Å, °)

*D*—H⋯*A*	*D*—H	H⋯*A*	*D*⋯*A*	*D*—H⋯*A*
O4—H4*A*⋯O1^ii^	0.77	1.96	2.718 (3)	169
O4—H4*B*⋯O3^iii^	0.73	1.96	2.685 (3)	177
O5—H5*A*⋯O5^iv^	0.80	2.02	2.803 (3)	167
O5—H5*B*⋯O4	0.71	2.43	3.088 (4)	157

Symmetry codes: (ii) 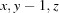; (iii) 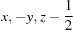; (iv) 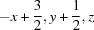.

**Table 3:** Experimental details

Crystal data	
Chemical formula	[Cu_2_(C_7_H_4_O_3_)_2_(H_2_O)_2_]·2H_2_O
*M*_r_	471.34
Crystal system, space group	Orthorhombic, *P* *b* *c* *n*
Temperature (K)	150
*a*, *b*, *c* (Å)	19.5028 (17), 5.0553 (4), 15.7573 (13)
*V* (Å^3^)	1553.6 (2)
*Z*	4
Radiation type	Mo *K*α
μ (mm^−1^)	2.80
Crystal size (mm)	0.17 × 0.09 × 0.02

Data collection	
Diffractometer	Bruker Kappa APEXII CCD
Absorption correction	Numerical (*SADABS*; Sheldrick, 2014 ▸)
*T*_min_, *T*_max_	0.669, 1.000
No. of measured, independent and observed [*I* > 2σ(*I*)] reflections	14169, 1799, 1186
*R*_int_	0.082
(sin θ/λ)_max_ (Å^−1^)	0.650

Refinement	
*R*[*F* ^2^ > 2σ(*F* ^2^)], *wR*(*F* ^2^), *S*	0.038, 0.098, 1.04
No. of reflections	1799
No. of parameters	120
H-atom treatment	H-atom parameters constrained
Δρ_max_, Δρ_min_ (e Å^−3^)	0.77, −0.58

Computer programs: *APEX2* (Bruker, 2007 ▸), *PEAKREF* (Schreurs, 2016 ▸), *Eval15* (Schreurs *et al.*, 2010 ▸), *SHELXT* (Sheldrick, 2015*a* ▸), *SHELXL2016* (Sheldrick, 2015*b* ▸), *PLATON* (Spek, 2009 ▸), *DRAWxtl* (Finger *et al.*, 2007 ▸) and *publCIF* (Westrip, 2010 ▸).

## supplementary crystallographic information

### Crystal data

**Table:**

[Cu_2_(C_7_H_4_O_3_)_2_(H_2_O)_2_]·2H_2_O	*D*_x_ = 2.015 Mg m^−^^3^
*M**_r_* = 471.34	Mo *K*α radiation, λ = 0.71073 Å
Orthorhombic, *P**b**c**n*	Cell parameters from 6768 reflections
*a* = 19.5028 (17) Å	θ = 2.1–27.5°
*b* = 5.0553 (4) Å	µ = 2.80 mm^−^^1^
*c* = 15.7573 (13) Å	*T* = 150 K
*V* = 1553.6 (2) Å^3^	Plate, brown
*Z* = 4	0.17 × 0.09 × 0.02 mm
*F*(000) = 952	

### Data collection

**Table:**

Bruker Kappa APEXII CCD diffractometer	1186 reflections with *I* > 2σ(*I*)
Radiation source: sealed tube	*R*_int_ = 0.082
φ and ω scans	θ_max_ = 27.5°, θ_min_ = 2.1°
Absorption correction: numerical (*SADABS*; Sheldrick, 2014)	*h* = −25→25
*T*_min_ = 0.669, *T*_max_ = 1.000	*k* = −6→6
14169 measured reflections	*l* = −16→20
1799 independent reflections	

### Refinement

**Table:**

Refinement on *F*^2^	Primary atom site location: heavy-atom method
Least-squares matrix: full	Secondary atom site location: difference Fourier map
*R*[*F*^2^ > 2σ(*F*^2^)] = 0.038	Hydrogen site location: difference Fourier map
*wR*(*F*^2^) = 0.098	H-atom parameters constrained
*S* = 1.04	*w* = 1/[σ^2^(*F*_o_^2^) + (0.0424*P*)^2^ + 2.4851*P*] where *P* = (*F*_o_^2^ + 2*F*_c_^2^)/3
1799 reflections	(Δ/σ)_max_ < 0.001
120 parameters	Δρ_max_ = 0.77 e Å^−^^3^
0 restraints	Δρ_min_ = −0.58 e Å^−^^3^

### Special details

**Table:**

Geometry. All esds (except the esd in the dihedral angle between two l.s. planes) are estimated using the full covariance matrix. The cell esds are taken into account individually in the estimation of esds in distances, angles and torsion angles; correlations between esds in cell parameters are only used when they are defined by crystal symmetry. An approximate (isotropic) treatment of cell esds is used for estimating esds involving l.s. planes.

### Fractional atomic coordinates and isotropic or equivalent isotropic displacement parameters (Å^2^)

**Table:**

	*x*	*y*	*z*	*U*_iso_*/*U*_eq_	
Cu1	0.500000	0.18416 (12)	0.250000	0.01968 (19)	
Cu2	0.500000	0.000000	0.500000	0.00951 (16)	
O1	0.56220 (14)	0.4466 (5)	0.30330 (15)	0.0123 (6)	
O2	0.52168 (14)	0.1699 (5)	0.39545 (15)	0.0124 (6)	
O3	0.55744 (13)	0.2318 (5)	0.56341 (15)	0.0116 (6)	
C1	0.56041 (19)	0.3678 (7)	0.3812 (2)	0.0091 (7)	
C2	0.60045 (18)	0.4988 (7)	0.4466 (2)	0.0087 (7)	
C3	0.59722 (19)	0.4210 (7)	0.5328 (2)	0.0095 (7)	
C4	0.6396 (2)	0.5632 (7)	0.5908 (2)	0.0139 (8)	
H4	0.639097	0.515779	0.649085	0.017*	
C5	0.6809 (2)	0.7660 (8)	0.5647 (2)	0.0135 (8)	
H5	0.708139	0.857335	0.605141	0.016*	
C6	0.6837 (2)	0.8409 (7)	0.4794 (2)	0.0128 (8)	
H6	0.712306	0.982514	0.461553	0.015*	
C7	0.64451 (19)	0.7064 (7)	0.4224 (2)	0.0117 (8)	
H7	0.646947	0.754349	0.364212	0.014*	
O4	0.57447 (14)	−0.0767 (5)	0.22503 (15)	0.0121 (6)	
H4A	0.568654	−0.200793	0.252177	0.018*	
H4B	0.571466	−0.118039	0.180993	0.018*	
O5	0.71893 (16)	0.1128 (6)	0.2774 (2)	0.0314 (8)	
H5A	0.730462	0.264763	0.277138	0.047*	
H5B	0.684633	0.112719	0.262689	0.047*	

### Atomic displacement parameters (Å^2^)

**Table:**

	*U*^11^	*U*^22^	*U*^33^	*U*^12^	*U*^13^	*U*^23^
Cu1	0.0208 (4)	0.0075 (3)	0.0308 (4)	0.000	−0.0139 (4)	0.000
Cu2	0.0158 (3)	0.0101 (3)	0.0026 (3)	−0.0037 (3)	−0.0009 (3)	0.0004 (2)
O1	0.0213 (15)	0.0107 (13)	0.0049 (11)	−0.0023 (11)	−0.0028 (11)	0.0029 (10)
O2	0.0217 (14)	0.0107 (12)	0.0048 (12)	−0.0056 (10)	0.0003 (10)	0.0010 (10)
O3	0.0193 (15)	0.0119 (12)	0.0037 (11)	−0.0058 (11)	−0.0006 (11)	0.0020 (10)
C1	0.0132 (18)	0.0091 (16)	0.0050 (16)	0.0025 (14)	0.0014 (14)	0.0024 (14)
C2	0.0111 (18)	0.0093 (16)	0.0056 (16)	0.0024 (15)	−0.0008 (14)	−0.0009 (14)
C3	0.0128 (19)	0.0099 (17)	0.0058 (16)	0.0024 (15)	−0.0010 (15)	−0.0002 (15)
C4	0.019 (2)	0.0144 (19)	0.0080 (17)	−0.0021 (16)	−0.0006 (16)	−0.0011 (15)
C5	0.0141 (19)	0.0181 (19)	0.0082 (17)	−0.0032 (16)	−0.0031 (15)	−0.0021 (15)
C6	0.0155 (19)	0.0097 (18)	0.0134 (19)	−0.0023 (15)	0.0017 (15)	−0.0031 (14)
C7	0.0152 (19)	0.0121 (18)	0.0078 (17)	0.0021 (15)	0.0000 (15)	0.0029 (15)
O4	0.0203 (14)	0.0121 (13)	0.0038 (11)	0.0013 (11)	−0.0017 (10)	0.0021 (10)
O5	0.0300 (18)	0.0267 (16)	0.0374 (18)	−0.0011 (15)	−0.0086 (15)	0.0095 (15)

### Geometric parameters (Å, º)

**Table:**

Cu1—O1	1.984 (2)	C2—C7	1.409 (5)
Cu1—O1^i^	1.984 (2)	C2—C3	1.416 (5)
Cu1—O4^i^	2.001 (3)	C3—C4	1.426 (5)
Cu1—O4	2.001 (3)	C4—C5	1.367 (5)
Cu1—O2	2.332 (2)	C4—H4	0.9500
Cu1—O2^i^	2.332 (2)	C5—C6	1.397 (5)
Cu2—O3^ii^	1.905 (2)	C5—H5	0.9500
Cu2—O3	1.905 (2)	C6—C7	1.361 (5)
Cu2—O2^ii^	1.905 (2)	C6—H6	0.9500
Cu2—O2	1.905 (2)	C7—H7	0.9500
O1—C1	1.292 (4)	O4—H4A	0.7679
O2—C1	1.274 (4)	O4—H4B	0.7271
O3—C3	1.322 (4)	O5—H5A	0.8007
C1—C2	1.452 (5)	O5—H5B	0.7079
			
O1—Cu1—O1^i^	96.07 (14)	O2—C1—O1	115.2 (3)
O1—Cu1—O4^i^	143.25 (10)	O2—C1—C2	123.5 (3)
O1^i^—Cu1—O4^i^	94.60 (11)	O1—C1—C2	121.3 (3)
O1—Cu1—O4	94.60 (11)	C7—C2—C3	119.5 (3)
O1^i^—Cu1—O4	143.25 (10)	C7—C2—C1	118.4 (3)
O4^i^—Cu1—O4	97.54 (15)	C3—C2—C1	122.0 (3)
O1—Cu1—O2	59.59 (9)	O3—C3—C2	125.2 (3)
O1^i^—Cu1—O2	123.20 (9)	O3—C3—C4	118.1 (3)
O4^i^—Cu1—O2	85.29 (9)	C2—C3—C4	116.7 (3)
O4—Cu1—O2	92.36 (9)	C5—C4—C3	121.8 (3)
O1—Cu1—O2^i^	123.20 (9)	C5—C4—H4	119.1
O1^i^—Cu1—O2^i^	59.59 (9)	C3—C4—H4	119.1
O4^i^—Cu1—O2^i^	92.37 (9)	C4—C5—C6	121.0 (4)
O4—Cu1—O2^i^	85.29 (9)	C4—C5—H5	119.5
O2—Cu1—O2^i^	176.45 (12)	C6—C5—H5	119.5
O3^ii^—Cu2—O3	180.0	C7—C6—C5	118.5 (3)
O3^ii^—Cu2—O2^ii^	92.62 (10)	C7—C6—H6	120.8
O3—Cu2—O2^ii^	87.38 (10)	C5—C6—H6	120.8
O3^ii^—Cu2—O2	87.38 (10)	C6—C7—C2	122.5 (3)
O3—Cu2—O2	92.62 (10)	C6—C7—H7	118.8
O2^ii^—Cu2—O2	180.0	C2—C7—H7	118.8
C1—O1—Cu1	100.4 (2)	Cu1—O4—H4A	108.8
C1—O2—Cu2	129.6 (2)	Cu1—O4—H4B	108.6
C1—O2—Cu1	84.86 (19)	H4A—O4—H4B	106.5
Cu2—O2—Cu1	145.44 (13)	H5A—O5—H5B	105.3
C3—O3—Cu2	126.8 (2)		
			
Cu2—O2—C1—O1	−176.6 (2)	C7—C2—C3—O3	179.2 (3)
Cu1—O2—C1—O1	1.0 (3)	C1—C2—C3—O3	−1.9 (6)
Cu2—O2—C1—C2	3.7 (5)	C7—C2—C3—C4	0.4 (5)
Cu1—O2—C1—C2	−178.8 (3)	C1—C2—C3—C4	179.3 (3)
Cu1—O1—C1—O2	−1.2 (3)	O3—C3—C4—C5	−178.3 (4)
Cu1—O1—C1—C2	178.6 (3)	C2—C3—C4—C5	0.5 (5)
O2—C1—C2—C7	176.4 (3)	C3—C4—C5—C6	−0.6 (6)
O1—C1—C2—C7	−3.4 (5)	C4—C5—C6—C7	−0.3 (6)
O2—C1—C2—C3	−2.5 (6)	C5—C6—C7—C2	1.3 (6)
O1—C1—C2—C3	177.7 (3)	C3—C2—C7—C6	−1.3 (5)
Cu2—O3—C3—C2	4.9 (5)	C1—C2—C7—C6	179.7 (4)
Cu2—O3—C3—C4	−176.3 (2)		

Symmetry codes: (i) −*x*+1, *y*, −*z*+1/2; (ii) −*x*+1, −*y*, −*z*+1.

### Hydrogen-bond geometry (Å, º)

**Table:**

*D*—H···*A*	*D*—H	H···*A*	*D*···*A*	*D*—H···*A*
O4—H4*A*···O1^iii^	0.77	1.96	2.718 (3)	169
O4—H4*B*···O3^iv^	0.73	1.96	2.685 (3)	177
O5—H5*A*···O5^v^	0.80	2.02	2.803 (3)	167
O5—H5*B*···O4	0.71	2.43	3.088 (4)	157

Symmetry codes: (iii) *x*, *y*−1, *z*; (iv) *x*, −*y*, *z*−1/2; (v) −*x*+3/2, *y*+1/2, *z*.
